# Correction: Analysis the patients’ careflows using process mining

**DOI:** 10.1371/journal.pone.0355134

**Published:** 2026-08-03

**Authors:** The PLOS One Editors

## Notice of republication

This article [1] was republished on July 13, 2026, to address an issue identified post-publication. Please download this article again to view the correct version.

The article’s Data Availability statement is updated to: The Data Availability statement is under discussion and will be provided in a forthcoming update to this article.
